# Review on Stress-Fractional Plasticity Models

**DOI:** 10.3390/ma15217802

**Published:** 2022-11-04

**Authors:** Pengfei Qu, Yifei Sun, Wojciech Sumelka

**Affiliations:** 1 School of Management Science and Engineering, Shandong Technology and Business University, Yantai 264005, China; 2 Key Laboratory of Geomechanics and Embankment Engineering of Ministry of Education, Hohai University, Nanjing 210098, China; 3 Institute of Structural Analysis, Poznan University of Technology, Piotrowo 5, 60-965 Poznan, Poland

**Keywords:** fractional derivative, fractional plasticity, nonassociated, state dependence

## Abstract

Fractional calculus plays an increasingly important role in mechanics research. This review investigates the progress of an interdisciplinary approach, fractional plasticity (FP), based on fractional derivative and classic plasticity since FP was proposed as an efficient alternative to modelling state-dependent nonassociativity without an additional plastic potential function. Firstly, the stress length scale (SLS) is defined to conduct fractional differential, which influences the direction and intensity of the nonassociated flow of geomaterials owing to the integral definition of the fractional operator. Based on the role of SLS, two branches of FP, respectively considering the past stress and future reference critical state can be developed. Merits and demerits of these approaches are then discussed, which leads to the definition of the third branch of FP, by considering the influences of both past and future stress states. In addition, some specific cases and potential applications of the third branch can be realised when specific SLS are adopted.

## 1. Introduction

Geomaterials, such as clay, sand, ballast and rock, are often encountered or used in practical engineering [1,2]. Before the designing and construction of infrastructure, site investigation was usually carried out, to have a full understanding of the mechanical properties of the underlying geomaterial. It was found that the constitutive responses of geomaterials were state-dependent and non-associated, due to the frictional nature [3,4,5,6]. The associated plasticity developed for metals could not be simply employed for modelling the stress and strain behaviour of geomaterials [7,8]. Instead, the non-associated plasticity within the framework of critical state soil mechanics was often suggested [9]. However, the classic non-associated plastic models required the incorporation of an additional plastic potential function and a state parameter, to capture the state-dependent non-associated behaviour of geomaterials, which inevitably resulted in the complexity of the developed model. Recently, nonconventional mechanical approaches using fractional calculus [10,11,12,13] have attracted increasing attention. Inspired by the fractional viscoplasticity (FVP) originally proposed by Sumelka [14,15], Sun and Sumelka [16], Lu et al. [17,18] and Qu [19,20] developed a series of fractional plasticity (FP) models, to solve this limitation. Without using an additional plastic potential function, the developed approach can be used to characterise the state-dependent non-associated stress-dilatancy behaviour of geomaterials.

In this study, a comprehensive introduction to the development and application of the FP for geomaterials will be provided, in terms of the role of SLS for carrying out the fractional differentiation. Three branches of the FP will be proposed and discussed. This study is intended to provide potential guidance for those who have an interest in this research branch of stress-fractional mechanics.

## 2. Progress in FP

The FP was originally established by incorporating the stress-fractional operator into the associated plasticity. It was inspired by the pioneering work of Sumelka [14,15] on the FVP. Nevertheless, these two types of research have differences with regard to the initial definition of the stress-fractional operator. According to Sumelka et al. [21,22], the FVP was developed based on the ‘short memory principle’, where the close virtual neighbourhood of a stress state ($\sigma_{ij}^{{}^{\prime }}$) (at a material point of interest) influences the fractional viscoplastic strain ($d\varepsilon_{ij}^{\mathrm{vp}}$) direction of the material, such that:

(1) $$d\varepsilon_{ij}^{\mathrm{vp}}=\Lambda \frac{{}_{\;\;\;\;\;a}^{\mathrm{RC}}D_{b}^{\alpha }f\left(\sigma_{ij}^{{}^{\prime }}\right)}{∥{}_{\;\;\;\;\;a}^{\mathrm{RC}}D_{b}^{\alpha }f\left(\sigma_{ij}^{{}^{\prime }}\right)∥}$$

where i,j = 1, 2, 3; Λ is the intensity of viscoplastic flow (provided as a material function, as in original Perzyna [23] approach); *D* indicates partial differential; *f* is yielding function; *a* and *b* denote the close virtual neighbourhood of a stress state ($\sigma_{ij}^{{}^{\prime }}$); α is the fractional-order, with α∈(n−1,n] and *n* the positive integer;   indicates the norm of a tensor. The superscript, RC, denotes the Riesz–Caputo fractional derivative, where in FVP it was defined by using the ‘short memory principle’ as:

(2) $${}_{\;\;\;\;\;a}^{\mathrm{RC}}D_{b}^{\alpha }f\left(\sigma_{ij}^{{}^{\prime }}\right)=\frac{1}{2}\left[{}_{a}^{C}D_{\sigma_{ij}^{{}^{\prime }}}^{\alpha }f\left(\sigma_{ij}^{{}^{\prime }}\right)+(-1{{)}^{n}}_{\sigma_{ij}^{{}^{\prime }}}^{\;\;C}D_{b}^{\alpha }f\left(\sigma_{ij}^{{}^{\prime }}\right)\right]$$

in which the superscript, C, indicates the Caputo fractional derivative; the definition of the Caputo fractional derivative can be found in the Appendix A. Note that ${}_{a}^{C}D_{\sigma_{ij}^{{}^{\prime }}}^{\alpha }f\left(\sigma_{ij}^{{}^{\prime }}\right)$ is the left-sided fractional derivative, whereas ${}_{\sigma_{ij}^{{}^{\prime }}}^{\;\;C}D_{b}^{\alpha }f\left(\sigma_{ij}^{{}^{\prime }}\right)$ is the right-sided fractional derivative.

Compared with FVP, the FP was developed based on the ‘long memory principle’, where the initial stress onset $\left(\sigma_{0}^{\prime }\right)$ or the targeted future stress $\left(\sigma_{cij}^{\prime }\right)$ influences the fractional plastic flow of the material at the current stress state. Thus, it is defined as:

(3) $$d\varepsilon_{ij}^{p}=d\lambda_{\;\;\;\;\;\;\;\sigma_{0}^{\prime }}^{C,\mathrm{RL}}D_{\sigma_{ij}^{\prime }}^{\alpha }f\left(\sigma_{ij}^{\prime }\right),\;\sigma_{ij}^{\prime }>\sigma_{0}^{\prime }$$

or

(4) $$\left\{\begin{array}{c} d\varepsilon_{ij}^{p}=d\lambda_{\;\;\;\;\;\;\sigma_{ij}^{{}^{\prime }}}^{C,\mathrm{RL}}D_{\sigma_{cij}^{{}^{\prime }}}^{\alpha }f\left(\sigma_{ij}^{{}^{\prime }}\right),\;\sigma_{cij}^{{}^{\prime }}>\sigma_{ij}^{{}^{\prime }}\\ d\varepsilon_{ij}^{p}=d\lambda_{\;\;\;\;\;\;\sigma_{cij}^{{}^{\prime }}}^{C,\mathrm{RL}}D_{\sigma_{ij}^{{}^{\prime }}}^{\alpha }f\left(\sigma_{ij}^{{}^{\prime }}\right),\;\sigma_{ij}^{{}^{\prime }}>\sigma_{cij}^{{}^{\prime }} \end{array}\right.$$

where the superscript (C, RL) indicates Caputo fractional derivative or Riemann–Liouville’s fractional derivatives; dλ is the non-negative plastic multiplier; $\sigma_{0}^{\prime }$ and $\sigma_{cij}^{\prime }$ are the integral limits. Equation (3) indicates the first type of the FP, here denoted as FP-n (n stands for nonassociated), which was adopted by researchers [17,19,24] for capturing the nonassociated plastic flow of granular soil, while Equation (4) indicates the second type of FP, here denoted as FP-sn (sn stands for state-dependent nonassociated), which was defined in [25] for modelling the state-dependent nonassociated behaviour of granular soil. It is noted that the FP-n based on Equation (3) assumes that the past loading history $\left(\sigma_{0}^{\prime }\to \sigma_{ij}^{\prime }\right)$ plays a role in the nonassociated plastic flow of geomaterial; the FP-sn based on Equation (4) assumes an effect of the future reference critical state, i.e., the distance $\left(\sigma_{ij}^{\prime }-\sigma_{cij}^{\prime }\right)$ from the current stress state $\sigma_{ij}^{{}^{\prime }}$ to the corresponding future reference critical state $\sigma_{cij}^{{}^{\prime }}$, on the current plastic flow direction of geomaterial. Note that the future reference critical state is a state which can be reached by soils subjected to sufficient shearing. This state was experimentally and numerically evidenced in many reported researches [3,4,6,7,26,27,28], and characterized by the critical state lines in the mean effective stress v.s. deviator stress space and the mean effective stress v.s. void ratio space. The current state is moving along the yield curve until reaching the critical state line. Although the FP was developed based on using fractional derivatives with power-law kernel, it can be also developed by other definitions using, for example, the exponential kernel, as long as it has analytical solutions of the yielding function. However, no matter which definition is used, the basic constitutive relation for FP-n and FP-sn should be the same.

Reformulating Equations (3) and (4), one can have a unified description for the FP as:

(5) $$d\varepsilon_{ij}^{p}=d\lambda \frac{\partial^{\alpha }f\left(\sigma_{ij}^{\prime },\hbar_{ij}\right)}{\partial \sigma_{ij}^{\prime \alpha }}$$

where $\hbar_{ij}$ denotes the hardening variable of the yielding function. Then, one needs to determine dλ for model application. Through applying the consistency condition at the yielding surface, one has:

(6) $$df=\frac{\partial f\left(\sigma_{kl}^{\prime },\hbar_{kl}\right)}{\partial \sigma_{kl}^{\prime }}d\sigma_{kl}^{\prime }+\frac{\partial f\left(\sigma_{kl}^{\prime },\hbar_{kl}\right)}{\partial \hbar_{kl}}d\hbar_{kl}=0$$

where the hardening variable $d\hbar_{kl}=\frac{\partial \hbar_{kl}}{\partial \varepsilon_{ab}^{p}}d\varepsilon_{ab}^{p}$. Substituting Equation (5) into Equation (6), one has:

(7) $$d\lambda =-\frac{\frac{\partial f\left(\sigma_{kl}^{{}^{\prime }},\hbar_{kl}\right)}{\partial \sigma_{kl}^{{}^{\prime }}}d\sigma_{kl}^{{}^{\prime }}}{\frac{\partial f\left(\sigma_{kl}^{{}^{\prime }},\hbar \right)\partial \hbar_{kl}\partial^{\alpha }f\left(\sigma_{ab}^{{}^{\prime }},\hbar_{ab}\right)}{\partial \hbar_{kl}\;\;\;\;\;\;\;\;\partial \varepsilon_{ab}^{p}\;\;\;\;\;\;\;\;\;\;\;\;{\partial \sigma_{ab}^{{}^{\prime }}}^{\alpha }}}$$

Then, substituting Equation (7) into Equation (5), one has the following constitutive relation for the FP:

(8) $$d\varepsilon_{ij}^{p}=\frac{1}{H}n_{ij}m_{kl}d\sigma_{kl}^{\prime }$$

where the hardening modulus (*H*), plastic flow tensor ($n_{ij}$), and plastic loading tensor ($m_{kl}$) can be derived as:

(9) $$H=\frac{-\frac{\partial f\left(\sigma_{kl}^{{}^{\prime }},\hbar_{kl}\right)\partial \hbar_{kl}\partial^{\alpha }f\left(\sigma_{ab}^{{}^{\prime }},\hbar_{ab}\right)}{\partial \hbar_{kl}\;\;\;\;\;\;\;\;\partial \varepsilon_{ab}^{p}\;\;\;\;\;\;\;\;\;\;\;\;{\partial \sigma_{ab}^{{}^{\prime }}}^{\alpha }}}{∥\frac{\partial^{\alpha }f\left(\sigma_{rs}^{{}^{\prime }},\hbar_{rs}\right)}{{\partial \sigma_{rs}^{{}^{\prime }}}^{\alpha }}∥∥\frac{\partial f\left(\sigma_{ct}^{{}^{\prime }},\hbar_{ct}\right)}{\partial \sigma_{ct}^{{}^{\prime }}}∥}$$

(10) $$n_{ij}=\frac{\frac{\partial^{\alpha }f\left(\sigma_{ij}^{\prime },\hbar_{ij}\right)}{\partial \sigma_{ij}^{\prime \alpha }}}{∥\frac{\partial^{\alpha }f\left(\sigma_{rs}^{\prime },\hbar_{rs}\right)}{\partial \sigma_{rs}^{\prime \alpha }}∥}$$

(11) $$m_{kl}=\frac{\frac{\partial f\left(\sigma_{kl}^{\prime },\hbar_{kl}\right)}{\partial \sigma_{kl}^{\prime }}}{∥\frac{\partial f\left(\sigma_{ct}^{\prime },\hbar_{ct}\right)}{\partial \sigma_{ct}^{\prime }}∥}$$

**Figure 1:**
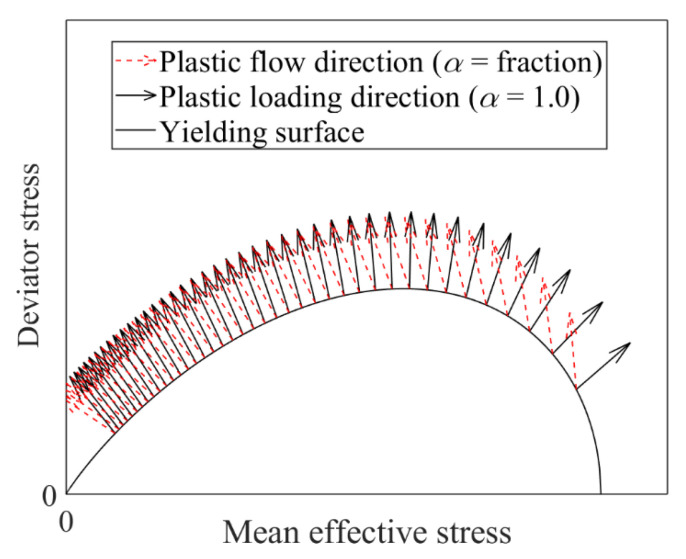
Schematic show of the loading and plastic flow directions.

Figure 1 modified from [29] schematically shows the plastic flow and loading directions calculated using Equations (10) and (11), where a deviation of the plastic flow direction from the plastic loading direction can be observed, indicating a nonassociated plastic flow rule in the developed FP. Based on Equation (8), a series of FP models were developed for the constitutive descriptions of different geomaterials. Depending on the definition of the adopted fractional operator, these FP models can be categorised into two branches, i.e., the ones considering the role of ‘past’ SLS [18,20,24] and the others considering the role of ‘future’ critical-state SLS [25]. These two branches will be respectively introduced in the next two sections.

### 2.1. FP-n: The Role of Past SLS

#### 2.1.1. Modelling of Soils

As indicated by Equation (3), compared with the previous works [30,31], the SLS is characterized by the length from current stress state to the reference stress state. Sun et al. [32] pointed out that in geotechnical engineering if one took the consolidation pressure instead of the zero-stress state as the initial stress state ($\sigma_{0}^{\prime }$), the developed model could predict a higher strain. However, this prediction difference could be reduced by tuning the values of some model parameters. For the sake of simplicity, $\sigma_{0}^{\prime }$ = 0 kPa was thus assumed for model derivation in most cases, cf. [18,19,20,33,34,35]. Through this assumption, the developed fractional plastic flow rule can be simple and yet flexible in constitutive modelling.

**Figure 2:**
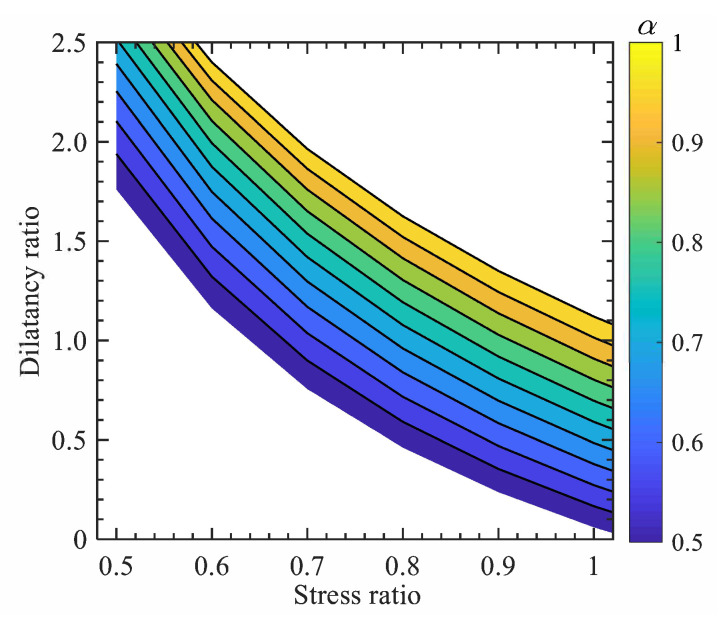
Effect of α on the stress-dilatancy relation.

Specifically speaking, to capture the stress-dilatancy behaviour of granular soil, the following fractional-order dilatancy ratio ($d_{g}$) based on the Modified Cam-clay (MCC) function was proposed [33]:

(12) $$d_{g}=\frac{{}_{0}D_{p^{\prime }}^{\alpha }\left(p^{\prime }\right)}{{}_{0}D_{q}^{\alpha }f\left(q\right)}=\frac{M^{2}-\left(1-\alpha /2\right)\left(\eta^{2}+M^{2}\right)}{\eta^{2-\alpha }}$$

where $p^{\prime }=1/3\sigma_{ij}^{\prime }\delta_{ij}$ and $q=\sqrt{3/2\left(\sigma_{ij}^{\prime }-p^{\prime }\delta_{ij}\right)\left(\sigma_{ij}^{\prime }-p^{\prime }\delta_{ij}\right)}$, are the mean effective stress and deviatoric stress, respectively; $\delta_{ij}$ is the Kronecker delta; *M* and η denote the critical-state and current-state stress ratios, respectively. Unlike other critical state parameters, the critical-state stress ratio (M) can be influenced by many factors, e.g., the particle shape [27], but it should not be affected by fines content [26,36] or shearing mode [28,37]. The effect of α on the stress-dilatancy behaviour of granular soil can be observed in Figure 2. It is found that with the increase of α, the dilatancy ratio at the same level of stress ratio increases, while with the increase of the stress ratio (η), the dilatancy ratio at the same α decreases.

It is worthwhile to mention that Equation (12) does not consider the dependence of stress-dilatancy on the material state in its current form unless a state-dependent parameter is introduced. However, state-dependent stress-dilatancy is a common phenomenon in granular soils, e.g., sand and rockfill, where the stress-dilatancy behaviour is determined by not only the current stress state but also the material state (i.e., void ratio, *e*, and pressure, $p^{\prime }$). To consider this, an empirical correlation of the fractional-order with the state parameter $\psi \left(=e-e_{c}\right)$ can be suggested, such that

(13) α=exp(−〈−Δψ〉)

where Δ is a material parameter; $e_{c}$ is the void ratio at the critical state; 〈〉 is Macauley brackets. Based on Equation (13), the state-dependent stress-dilatancy or plastic flow direction can be captured. There are two chances for Equation (12) to be equal to zero: one is at the phase transformation state where $d_{g}$ = 0, the other is at the critical state where η=M,ψ=0 and α = 1, which ensures that Equation (12) conforms to the basic restrictions of the Critical State Soil Mechanics (CSSM) [38].

Note that if a constant α was used, Equation (12) would either overestimate or underestimate the critical-state strength of the material, because, at the critical state, $d_{g}$ = 0, such that Equation (12) can be solved as:

(14) $$\eta_{c}=\sqrt{\frac{\alpha }{2-\alpha }}M$$

where $\eta_{c}$ indicates the calculated critical-state stress ratio. In critical state soil mechanics, $\eta_{c}=M$ should always exist at the critical state. However, this can only be true if α=1 at the critical state or one uses a different constant instead of *M* in Equation (12). The latter option was introduced by Liang, et al. [39] and Lu, et al. [17] to consider the effect of multiaxial loading on soft soils, e.g., clay, where they developed the multiaxial stress-dilatancy relation by using a well-established characteristic stress concept, such that Equation (12) can be reformulated as [40]:

(15) $$d_{g}=\frac{{}_{0}D_{{\tilde{p}}^{\prime }}^{\alpha }f\left({\tilde{p}}^{\prime }\right)}{{}_{0}D_{\tilde{q}}^{\alpha }f\left(\tilde{q}\right)}=\frac{N^{2}-\left(1-\alpha /2\right)\left({\tilde{\eta }}^{2}+N^{2}\right)}{{\tilde{\eta }}^{2-\alpha }}$$

where ${\tilde{p}}^{\prime }$, $\tilde{q}$ and $\tilde{\eta }$ are the characteristic stress components of $p^{\prime }$, *q* and η; *N* is a material parameter, different from *M* in the original FP model. Then, $\eta_{c}=M$ at the critical state can be guaranteed via a constant fractional-order shown in Equation (14), i.e., $\eta_{c}=\sqrt{\alpha /(2-\alpha )}N$. To consider the dependence of dilatancy on material state, a dependence of $d_{g}$ on the state parameter, for example, the relation in Equation (13) may be further introduced. However, there is one other option: that is to use the stress ratio at the phase transformation state, i.e., $M_{pt}$, to determine the fractional order as suggested by Liang, et al. [40]. As suggested by Nguyen and his coworkers [26,27,28,36,37], the phase-transformation-state parameter ($M_{pt}$) and strain hardening parameter ($M_{p}$) are also a function of *M* and the state parameter.

**Figure 3:**
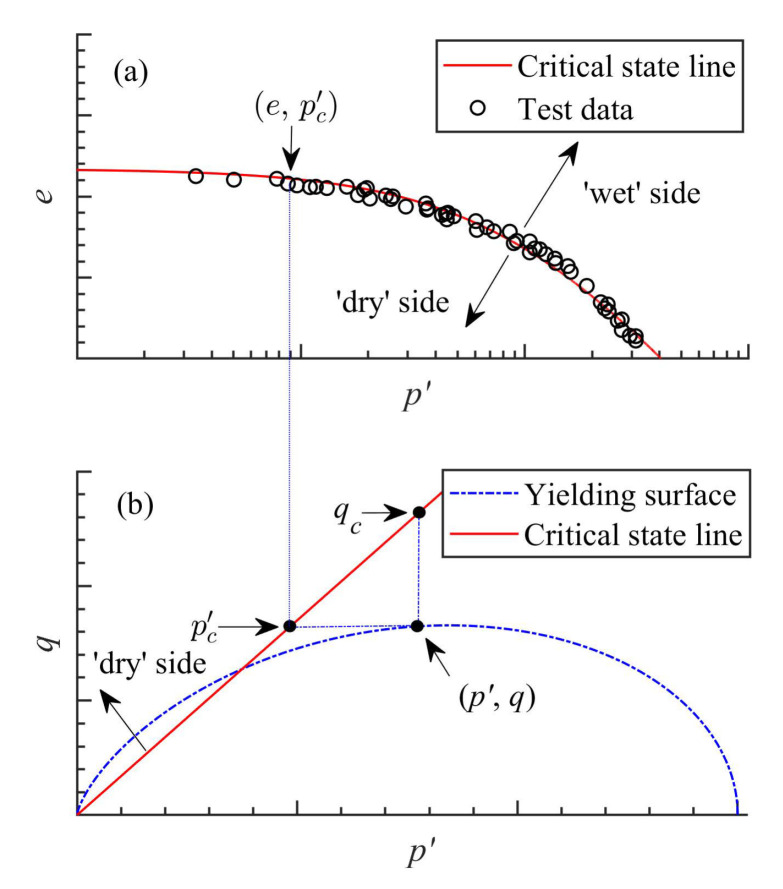
Relative position between current state and critical state in the (**a**) $e-p^{\prime }$ plane and (**b**) $p^{\prime }-q$ plane (data cited from Verdugo and Ishihara (1996)).

Despite the above successful applications, the FP-n models based on MCC function usually predicted much higher dilatancy for sand at the same stress level when compared to the corresponding test data [41]. This can be attributed to the larger elastic region of the MCC yielding surface at the ‘dry’ side (Figure 3 modified from [42]) of the critical state line in the $p^{\prime }-q$ plane [43]. For modelling sand, the original Cam-clay (CC) function [38] with reduced elastic region seems to work better. A new fractional stress-dilatancy relation based on the original CC function can be proposed by using the RL definition:

(16) $$d_{g}=\left\{\left[f_{d}\left(2\right)-f_{d}\left(2-\alpha \right)\right]M-\eta \right\}{\left|\eta \right|}^{\alpha -1}\frac{1}{\alpha }+\left(\frac{1}{\alpha }-1\right){\left|\eta \right|}^{\alpha }$$

where $f_{d}$ denotes the digamma function, which can be defined as $f_{d}=D^{1}\left(\ln\Gamma \right)$, with Γ the gamma function. It is easily found that $d_{g}=0$ at the critical state. However, at the phase transformation state, $d_{g}=0$ will result in a much complex condition for determining the fractional order from laboratory test data. For example, iteration should be required for parameter identification. Therefore, from the perspective of practical application, one may ask if a simplified version of the CC-based fractional dialtancy equation can be suggested, which can lead to a much easier way, i.e., directly measuring from test data, to determine the fractional order. In fact, during model development, the RL derivatives of constants can be omitted due to its limited influence on the dilatancy equation [44]. Because such influence can be compensated through further calibration of model parameters, e.g., the fractional order. Thus, a modified fractional stress-dilatancy relation for granular soil and soil-structure interface can be derived as:

(17) $$d_{g}=\left\{\left[f_{d}\left(2\right)-f_{d}\left(2-\alpha \right)\right]M-\eta \right\}{\left|\eta \right|}^{\alpha -1}$$

In addition, one can also derive Equation (17) by using the Caputo definition, as shown in [45]. Through such simplification, the RL definition and Caputo definition can lead to the same expression of $d_{g}$. To consider the state dependence, the fractional order can be also correlated to the state parameter via Equation (13). Equations (16) and (17) conform to the CSSM, as $d_{g}=0$ at both the phase transformation state and critical state.

#### 2.1.2. Modelling of Rocks

**Figure 4:**
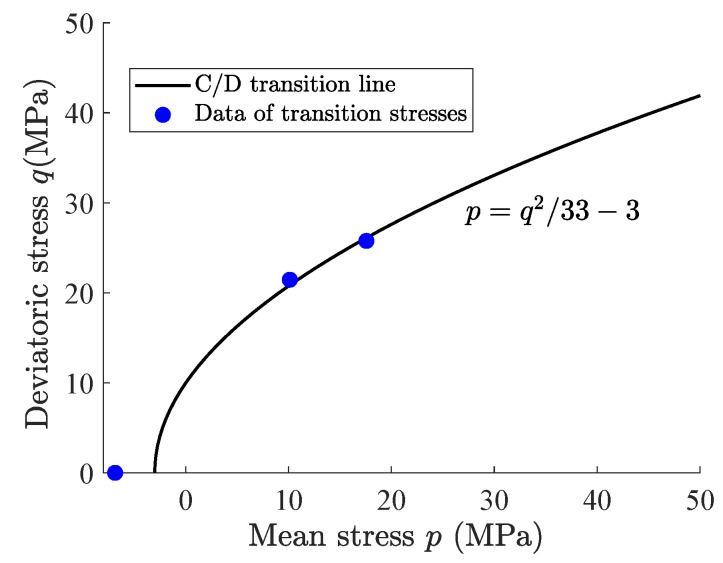
Compressibility/dilation (C/D) boundary of claystone subjected to conventional triaxial compression.

In addition to the application of FP-n in modelling granular or soft soils, several attempts have been also made to capture the stress-strain behaviours of rocks [19,20,46,47] and rock-like materials [18]. In these applications, different constitutive models with a fractional plastic flow rule were proposed based on the diverse problems that were focused on. For the purpose of describing the volumetric compression/dilation transition phenomenon of soft and hard rocks, Qu et al. [19] developed an elastoplastic model with fractional-order plastic flow where a unified hardening/sofening function $\kappa_{p}$ was proposed as follows:

(18) $$\begin{array}{ccc} & \kappa_{p}=\kappa_{p}^{0}+\left(1-\kappa_{p}^{0}\right)\frac{\Pi \xi }{\Pi +\xi^{\Pi }-1}, & \xi =\gamma^{p}/\gamma_{c}^{p}\; \end{array}$$

with

(19) $$\gamma^{p}=\int \sqrt{\frac{2}{3}de_{ij}^{p}de_{ij}^{p}},\;e_{ij}^{p}=\varepsilon_{ij}^{p}-\frac{1}{3}\mathrm{tr}\left(\varepsilon_{}^{p}\right)\delta_{ij}$$

in which $\varepsilon_{ij}^{p}$ denotes the plastic part of total strain $\varepsilon_{ij}$; $\gamma^{p}$ is equivalent plastic shear strain; $\gamma_{c}^{p}$ indicates the generalized plastic shear strain at peak stress; Π>1 represents the model parameter; $\kappa_{p}^{0}$ means the initial value corresponding to $\gamma^{p}=0$. Moreover, the maximum value $\kappa_{p}=1$ is obtained at the critical state $\gamma^{p}=\gamma_{c}^{p}$. To calibrate the fractional order α, Qu et al. [19] derived the formulation:

(20) $$-\frac{\partial f}{\partial \sigma_{ij}}D_{ijkl}d\varepsilon_{kl}A\kappa_{p}p^{1-\alpha }=\left(\frac{\partial f}{\partial \sigma_{ij}}D_{ijmn}\frac{\partial^{\alpha }f}{\partial \sigma_{mn}^{\alpha }}-\frac{\partial f}{\partial \kappa_{p}}\frac{\partial \kappa_{p}}{\partial \gamma_{p}}\frac{\partial^{\alpha }f}{\partial q^{\alpha }}\right)\delta_{rs}D_{rskl}^{-1}d\varepsilon_{kl}\Gamma \left(2-\alpha \right)$$

where *A* defines the friction coefficient; $D_{ijkl}$ is the fourth-order elasticity tensor; *p* and *q* are the mean stress and deviatoric stress, respectively. In the process of determining α, compressibility/dilation boundary $d\varepsilon_{v}=0$ of claystone subjected to conventional triaxial compression tests was employed and plotted in Figure 4.

**Figure 5:**
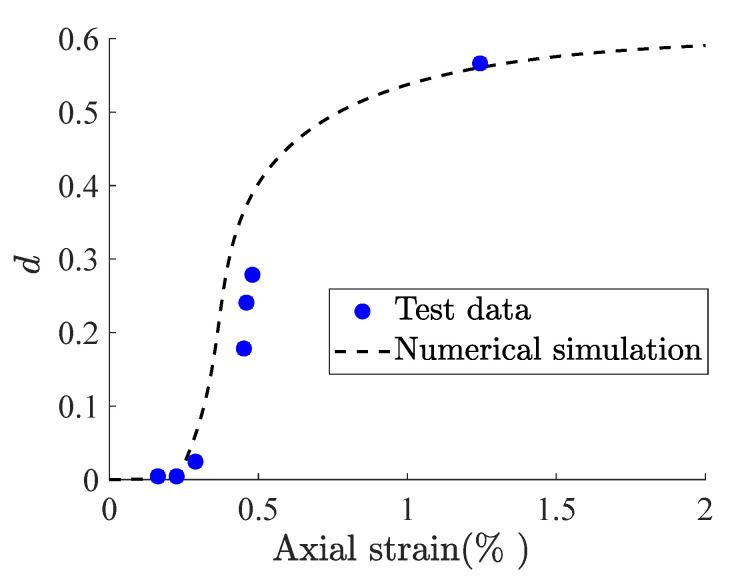
Numerical simulation of damage evolution versus axial strain under triaxial compression of $(\sigma_{3}$ = 10 MPa).

Note that the influence of micro-crack growth on plastic volume was not considered in [19]. Aiming to provide a new insight for investigating the complicated effect of plastic flow direction on damage evolution, Qu and Zhu [48] take the following damage evolution function $G_{d}$:

(21) $$G_{d}\left(\varepsilon^{p},d\right)=\;d-d_{c}\left[1-\exp\left(\upsilon \varepsilon_{v}^{p}\right)\right]=0$$

with $d_{c}$ being the asymptotic damage value in the residual stage, and υ indicates the material parameter controlling the velocity of the damage growth. Note that the variation of plastic volumetric strain $\varepsilon_{v}^{p}$ is related to the fractional order α as demonstrated in the following relation:

(22) $$d\varepsilon_{v}^{p}=\Lambda \frac{\partial^{\alpha }f}{\partial p^{\alpha }}$$

with *p* denoting the mean stress. As such, the numerical simulation of Beishan granite subjected to the confining pressure of 10MPa is displayed in Figure 5. It can be observed from Figure 5 that the developed fractional plastic damage model has the potential to reproduce the damage evolution under the loading process. Moreover, it can be found from [48] that the fractional order α plays a critical role in the damage growth. To further account for the influence of the fractional plastic flow on the micromechanics for quasi-brittle rocks, a friction criterion regarding local stresses was adopted as follows [20]:

(23) $$f\left(\sigma^{c}\right)=∥s^{c}∥-\tilde{A}p^{c}\leq 0$$

with

(24) $$\begin{array}{c} s^{c}=s-\frac{1}{\gamma_{2}\omega }2\mu^{m}\Gamma ,\;\;\;p^{c}=p+\frac{1}{\gamma_{1}\omega }k^{m}\beta \end{array}$$

where $p^{c}$ and $s^{c}$ denote the hydrostatic part and the deviatoric part of the local stress $\sigma^{c}$, respectively; $\tilde{A}$ is the generalized friction coefficient; s and *p* means the macroscopic deviatoric stress; $k^{m}$ and $\mu^{m}$ represent the bulk and shear moduli of the matrix, respectively; ω indicates microscopic damage internal variable; Γ and $\beta =\varepsilon^{p}:\delta$ describe the relative slip degree between microcrack surfaces and the degree of microcrack’s opening, respectively. $\gamma_{1}$ and $\gamma_{2}$ associated with the Poisson’s ratio of the solid matrix $\nu^{m}$ and can be written as:

(25) $$\gamma_{1}=\frac{16}{9}\frac{1-{\left(\nu^{m}\right)}^{2}}{1-2\nu^{m}},\;\;\;\gamma_{2}=\frac{32}{45}\frac{\left(1-\nu^{m}\right)\left(5-\nu^{m}\right)}{2-\nu^{m}}$$

**Figure 6:**
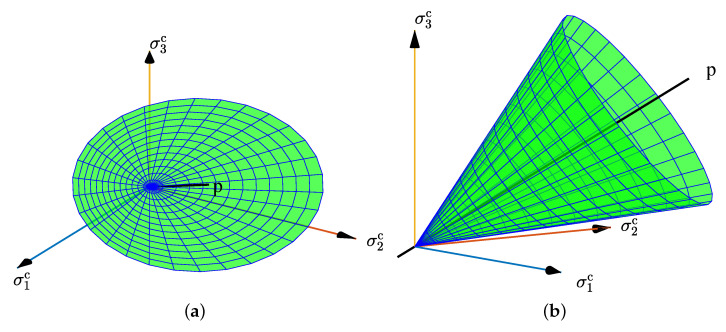
Yield surface in the local stress space: (**a**) front view, (**b**) lateral view.

Based on Equation (23), the yield surface in the local stress space can be given in Figure 6, which is a conical surface with the diagonal of the space as the axis.

**Figure 7:**
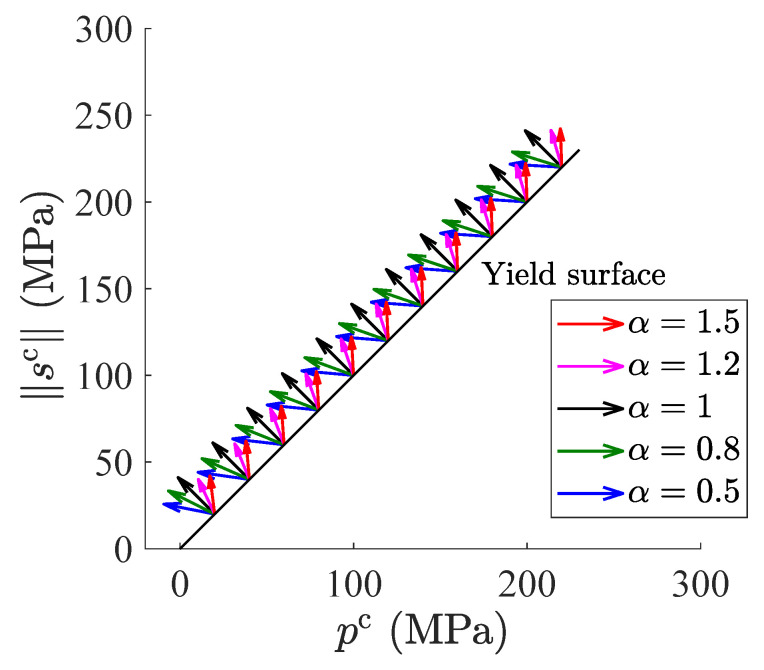
Influence of the fractional order on the plastic flow direction in $p^{c}-∥s^{c}∥$ plane with η=1.

Figure 7 shows the influence of the fractional order α on plasctic flow with the case of η=1 where dotted arrows represent the orthogonal direction and the solid arrows denote fractional plastic flow direction. It can be observed from Figure 7 that the fractional order brings a significant influence on the plasctic flow direction, especially under the high hydrostatic pressure. In Figure 7, the decrease of α results in a larger deviation from the orthogonal direction in the case of α<1. In Figure 7, the deviation from the loading direction is larger with an increase of α in the case of α>1. Note that when α=1, the fractional plastic flow direction degenerates to the classical associated plastic flow as shown in Figure 7. Hence, it is found that the change of the fractional order can capture plastic flow direction more flexibly without the additional plastic potentical.

**Figure 8:**
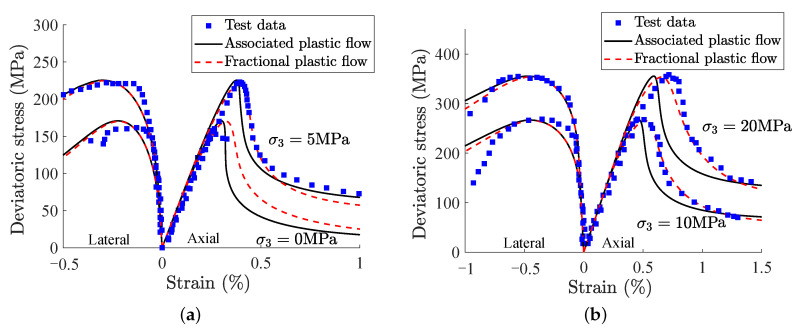
Comparisons between test data and the model predictions of Beishan granite under triaxial compression tests with different confining pressures:(**a**) $\sigma_{3}$ = 0 and 5 MPa, (**b**) $\sigma_{3}$ = 10 and 20 MPa.

In [20], comparisons between test data and simulation results on Beishan granite under the confining pressure of $\sigma_{3}=0,5,10$ and 20MPa are displayed in Figure 8. Numerical results of the fractional model are in good agreement with test data. By comparing the traditional associated model and the fractional model, it can be found that the fractional model have better performance on reproducing the main features of mechanical behaviors of Beishan granite, especially in the softening phase.

In addition, Li et al. [46] established a fractional constitutive model of soft rock considering temperature effect where model parameter *m* related to dilatancy characteristics was introduced. In this model, the relation between the fractional order α and similarity factor *R* is given by:

(26) $$\alpha =\frac{2R^{2m}}{1+R^{2m}}$$

Based on the microstructure of porous matrix-inclusion, Shen et al. [47] developed an elastoplastic damage constitutive model with a fractional plastic flow where the yield criterion can be applied as follows:

(27) $$\begin{array}{cc} f= & \frac{\frac{1+2\ell /3}{{\tilde{A}}^{2}}+\frac{2}{3}\rho \left(\frac{3\ell }{2{\tilde{A}}^{2}}-1\right)}{\frac{4{\tilde{A}}^{2}-12\ell -9}{6{\tilde{A}}^{2}-13\ell -6}\rho +1}q^{2}+\left(\frac{3\ell }{2{\tilde{A}}^{2}}-1\right)p^{2}+2\left(1-\ell \right)hp\\ \; & -\frac{3+2\ell +3\ell \rho }{3+2\ell }{\left(1-\ell \right)}^{2}h^{2}=0 \end{array}$$

where *ℓ* and ρ represent the volume fraction of pores and the volume fraction of inclusions, respectively; *h* is the hydrostatic tensile strength. This study [47] shows that when considering the material microstructure information including the porosity, the inclusion and the solid phase, the introduction of the fractional plasticity is still effective.

To better simulate the direction and magnitude of $d\varepsilon_{ij}^{p}$ for rock-like material, i.e., concrete, Lu et al. [18] proposed a three-dimensional fractional elastoplastic constitutive model in which the expression of fractional plastic flow direction is as follows:

(28) $$n={\left[{\left({\tilde{n}}^{\alpha }\right)}^{T}:{\left(\frac{\partial \tilde{p}}{\partial \tilde{\sigma }},\frac{\partial \tilde{q}}{\partial \tilde{\sigma }},\frac{\partial \tilde{\theta }}{\partial \tilde{\sigma }}\right)}^{T}:\frac{\partial \tilde{\sigma }}{\partial \sigma }\right]}^{T}$$

where $\tilde{\sigma }$ is the transformed stress tensor; $\tilde{p}$, $\tilde{q}$ and $\tilde{\theta }$ are the hydrostatic pressure, the deviatoric stress and the Lode angle in the transformed stress space, respectively. The fractional gradient of yield function ${\tilde{n}}^{\alpha }$ in the transformed stress space can be expressed as:

(29) $${\tilde{n}}^{\alpha }={\left(\frac{\partial^{\alpha_{1}}f}{\partial {\tilde{p}}^{\alpha_{1}}},\frac{\partial^{\alpha_{2}}f}{\partial {\tilde{q}}^{\alpha_{2}}},\frac{\partial^{\alpha_{3}}f}{\partial {\tilde{\theta }}^{\alpha_{3}}}\right)}^{T}={\left(\frac{\partial^{\alpha }f}{\partial {\tilde{p}}^{\alpha }},\frac{\partial^{\alpha }f}{\partial {\tilde{q}}^{\alpha }},\frac{\partial^{\alpha }f}{\partial {\tilde{\theta }}^{\alpha }}\right)}^{T}$$

in which $\alpha_{1}=\alpha_{2}=\alpha_{3}=\alpha$ for the simplification of the developed model. Finally, the corresponding stress-dilatancy relationship can be obtained, such that:

(30) $$d_{g}=-\frac{\left(\frac{\partial^{\alpha }f}{\partial {\tilde{p}}^{\alpha }},\frac{\partial^{\alpha }f}{\partial {\tilde{q}}^{\alpha }},\frac{\partial^{\alpha }f}{\partial {\tilde{\theta }}^{\alpha }}\right){\left(\frac{\partial \tilde{p}}{\partial q},\frac{\partial \tilde{q}}{\partial q},\frac{\partial \tilde{\theta }}{\partial q}\right)}^{T}}{\left(\frac{\partial^{\alpha }f}{\partial {\tilde{p}}^{\alpha }},\frac{\partial^{\alpha }\tilde{f}}{\partial {\tilde{q}}^{\alpha }},\frac{\partial^{\alpha }f}{\partial {\tilde{\theta }}^{\alpha }}\right){\left(\frac{\partial \tilde{p}}{\partial p},\frac{\partial \tilde{q}}{\partial p},\frac{\partial \tilde{\theta }}{\partial p}\right)}^{T}}$$

Together with damage feature of concrete materical, a 3D non-orthogonal plastic damage model is developed in [49] where α can be obtained based on the following equation of phase transformation:

(31) $$d\varepsilon_{v}^{p}=d\lambda {\tilde{p}}^{-\alpha }\left[\frac{a_{0}}{\Gamma (1-\alpha )}+\frac{a_{1}\Gamma \left(2\right)\tilde{p}}{\Gamma (2-\alpha )}+\frac{a_{2}\Gamma \left(3\right){\tilde{p}}^{2}}{\Gamma (3-\alpha )}+\frac{a_{3}\Gamma \left(4\right){\tilde{p}}^{3}}{\Gamma (4-\alpha )}+\frac{a_{4}\Gamma \left(5\right){\tilde{p}}^{4}}{\Gamma (5-\alpha )}\right]=0$$

where $a_{k}\left(k=0,1,2,3,4\right)$ are coefficients of power functions for $\tilde{p}$. Subsequently, Lu et al. [50] developed a cohesion-friction combined hardening plastic model of concrete based on the fractional flow rule. Moreover, this model is implemented with the help of an open-source user defined material subroutine UMAT in the framework of the implicit return mapping algorithm.

#### 2.1.3. Numerical Schemes

Integration algorithms significantly influence computation accuracy and efficiency in the process of the implementation of constitutive equations. For the fractional model as presented in [49], the Next Increment Corrects Error [51] approach were adopted where the workflow of the NICE algorithm can be summarized in Algorithm 1. In this Algorithm, *n* and n+1 are the current step and the previous step; $\bar{\sigma }$ is the effective stress; ${\bar{\sigma }}^{\mathrm{trial}\;}$ denotes the trial stress; $D_{0}$ indicates the undamaged elastic stiffness matrix; r refers to the direction of plastic strain increment.
**Algorithm 1:** Flowchart of the NICE algorithm for the fractional model
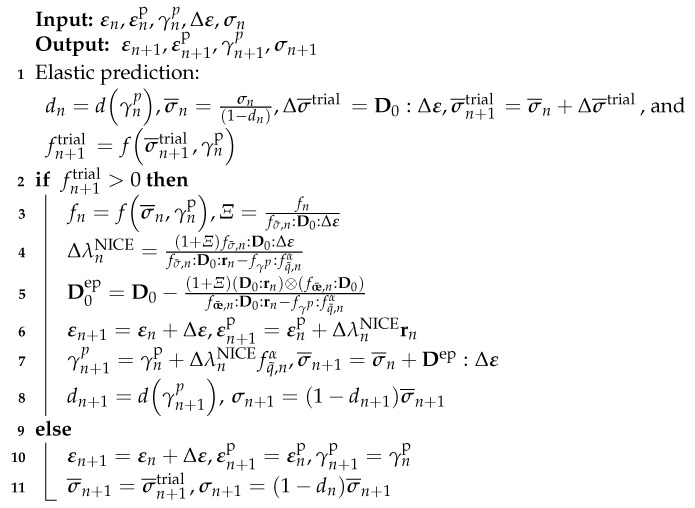


**Figure 9:**
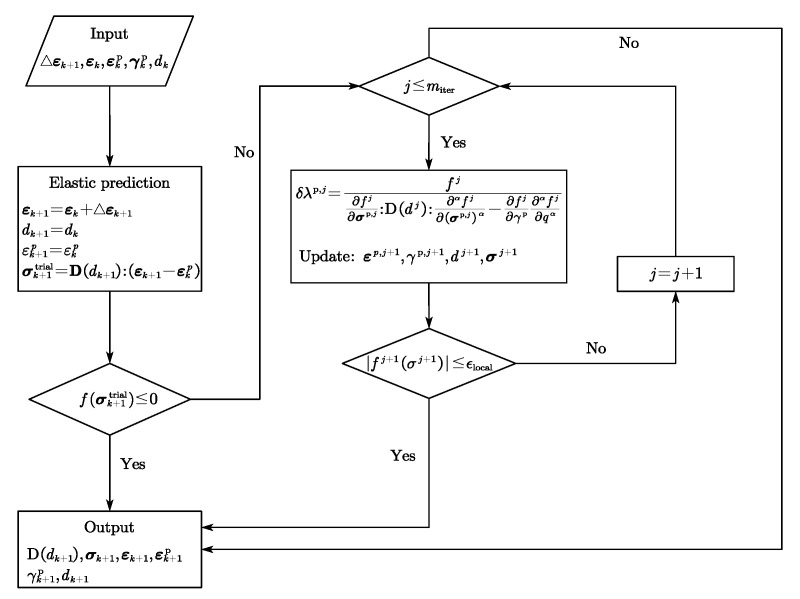
Flowchart of SRM algorithm.

**Figure 10:**
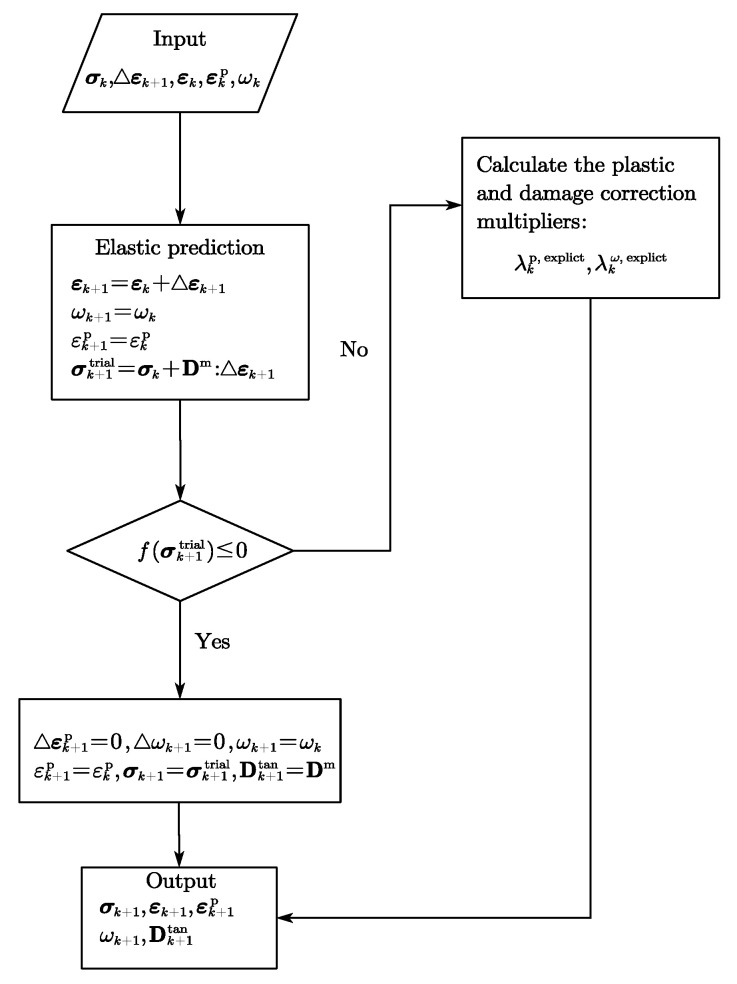
Flowchart of the explicit return mapping algorithm.

In addition, Qu and Zhu [48] proposed a semi-implicit return mapping (SRM) algorithm for the implementation of a novel fractional plastic damage model as illustrated in Figure 9. Aiming to more efficiently conduct a micromechanics-based fractional frictional damage model, an explicit return mapping algorithm was put forward in [20] and is given in Figure 10. Furthermore, it can be found that the numerical solutions are consistent with the analytical ones when increment step is enough large. Compared to the plasticity-damage decoupling correction (PDDC) algorithm proposed by [52], the explicit return mapping algorithm has a better performance in computational efficiency.

### 2.2. FP-sn: The Role of Future Reference Critical State

In this section, an introduction of the FP-sn models based on Equation (4) is made. It was observed in experimental tests that the volumetric dilatancy of soils, e.g., sand and over-consolidated clay, depends on not only the current state ($e,p^{\prime }$) but also the distance ($e-e_{c}$ or $p^{\prime }-p_{c}^{\prime }$) from current state to future reference critical state ($e_{c},p_{c}^{\prime }$).

**Figure 11:**
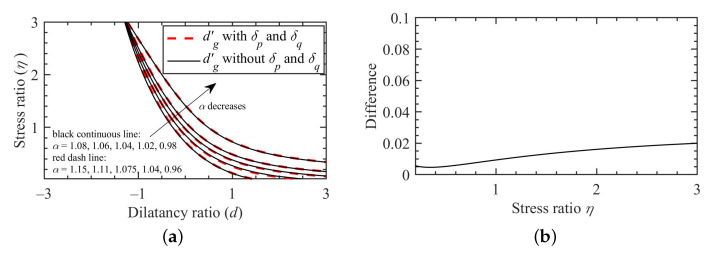
Predicted dilatancy ratios with and without $\delta_{p}$ and $\delta_{q}$: (**a**) dilatancy line, (**b**) mean difference between predicted $d^{\prime }g$ with and without $\delta_{p}$ and $\delta_{q}$.

After revisiting the CSSM, one can find that soils under shearing would finally reach the critical state represented by the critical-state void ratio ($e_{c}$), mean effective stress ($p_{c}^{\prime }$) and deviator stress ($q_{c}$). Here, $p_{c}^{\prime }=1/3\sigma_{cij}^{\prime }\delta_{ij}$ and $q_{c}=\sqrt{3/2\left(\sigma_{cij}^{\prime }-p_{c}^{\prime }\delta_{ij}\right)\left(\sigma_{cij}^{\prime }-p_{c}^{\prime }\delta_{ij}\right)}$. Then, it can be assumed that the future critical-state stresses ($p_{c}^{\prime },q_{c}$) can serve as the integral limit ($\sigma_{cij}^{\prime }$) in Equation (4). Substituting the MCC function into Equation (4) with RL and Caputo derivatives, one can obtain the following state-dependent stress-dilatancy relations for soil:

(32) $$d_{g}^{{}^{\prime }}=\frac{{}_{\;\;\;\;p_{c}^{{}^{\prime }}}^{\;\;\mathrm{RL}}D_{p^{{}^{\prime }}}^{\alpha }f\left(p^{{}^{\prime }}\right)}{{}_{\;\;\;\;q}^{\mathrm{RL}}D_{q_{c}}^{\alpha }f\left(q\right)}=\frac{{}_{\;\;\;\;p_{}^{{}^{\prime }}}^{\;\;\mathrm{RL}}D_{p_{c}^{{}^{\prime }}}^{\alpha }f\left(p^{{}^{\prime }}\right)}{{}_{\;\;\;\;q_{c}}^{\mathrm{RL}}D_{q}^{\alpha }f\left(q\right)}={\left|M\right|}^{1+\alpha }\frac{\left(p^{{}^{\prime }}-p_{c}^{{}^{\prime }}\right)+\left(2-\alpha \right)\left(p_{c}^{{}^{\prime }}-p_{0}^{{}^{\prime }}/2\right)+\delta_{p}}{\left(q-q_{c}\right)+\left(2-\alpha \right)q_{c}+\delta_{q}}$$

(33) $$d_{g}^{{}^{\prime \prime }}=\frac{{}_{\;\;\;\;p_{c}^{{}^{\prime }}}^{\;\;\;\;C}D_{p^{{}^{\prime }}}^{\alpha }f\left(p^{{}^{\prime }}\right)}{{}_{\;\;\;\;q}^{\;\;\;\;C}D_{q_{c}}^{\alpha }f\left(q\right)}=\frac{{}_{\;\;\;\;p_{}^{{}^{\prime }}}^{\;\;\;\;C}D_{p_{c}^{{}^{\prime }}}^{\alpha }f\left(p^{{}^{\prime }}\right)}{{}_{\;\;\;\;q_{c}}^{\;\;\;\;C}D_{q}^{\alpha }f\left(q\right)}={\left|M\right|}^{1+\alpha }\frac{\left(p^{\prime }-p_{c}^{\prime }\right)+\left(2-\alpha \right)\left(p_{c}^{\prime }-p_{0}^{\prime }/2\right)}{\left(q-q_{c}\right)+\left(2-\alpha \right)q_{c}}$$

where $p_{0}^{\prime }=\left[{\left(\eta /M\right)}^{2}+1\right]p^{\prime }$, is the size of the MCC yielding surface; $\delta_{p}=\left[p_{0}^{{}^{\prime }}-\left(p^{{}^{\prime }}+\right.\right.\left.\left.p_{c}^{{}^{\prime }}\right)\right]\left(2-\alpha \right)\left(1-\alpha \right)/2$ and $\delta_{q}=\left(q+q_{c}\right)\left(2-\alpha \right)\left(\alpha -1\right)/2$. Comparison between Equations (32) and (33) shows that there appears two additional items, i.e., $\delta_{p}$ and $\delta_{q}$, when using the RL definition. However, further analysis shown in Figure 11a can show that the influence of such two items on soil dialtancy can be compensated by tuning the value of fractional order. A very small difference between $d_{g}^{{}^{\prime }}$ with $\delta_{p}$ and $\delta_{q}$ and $d_{g}^{{}^{\prime \prime }}$ without $\delta_{p}$ and $\delta_{q}$ can be observed in Figure 11b, if a proper fractional order is used. Therefore, for practical application, the contributions from $\delta_{p}$ and $\delta_{q}$ were not considered through the omission of RL derivatives of constants. For the sake of simplicity, a unified $d_{g}$ is thus suggested, such that:

(34) $$d_{g}={\left|M\right|}^{1+\alpha }\frac{\left(p^{\prime }-p_{c}^{\prime }\right)+\left(2-\alpha \right)\left(p_{c}^{\prime }-p_{0}^{\prime }/2\right)}{\left(q-q_{c}\right)+\left(2-\alpha \right)q_{c}}$$

which also facilitates the calibration of model parameters directly from laboratory test data.

Moreover, the critical-state deviator stress ($q_{c}$) in Equations (32) and (33) can be calculated by checking the geometric position of the current stress and critical-state stress shown in Figure 3, such that:

(35) $$q_{c}=q+M\left(p^{\prime }-p_{c}^{\prime }\right)$$

while the critical-state mean effective pressure can be calculated using the critical state line shown in Figure 3a, such that:

(36) $$p_{c}^{\prime }=g\left(e\right)$$

where g(e) is a function describing the critical state line of soil in the $e-p^{\prime }$ plane. g(e) is determined by fitting the critical-state data points. There are different available formulae for g(e), but no matter which formula is used, a unique relation with $p_{c}^{{}^{\prime }}$ can be provided. For example, the g(e) for Toyoura sand [41] shown in Figure 3a can be expressed as:

(37) $$p_{c}^{\prime }=p_{r}\exp\left(\frac{e_{\Gamma }-e}{\lambda }\right)-p_{s}$$

where $p_{r}$ = 1 kPa, is the unit pressure for normalisation; $e_{\Gamma }$ and λ are material parameters; $p_{s}$ is the shift stress, describing the effect of particle breakage on the downward bending of the critical state line shown in Figure 3a.

It can be found from Equation (34) that $d_{g}$ also has two chances to reach zero: one is at the phase transformation state with a typical value of the fractional, the other is at the critical state with $p^{{}^{\prime }}=p_{c}^{{}^{\prime }}$ and $q=q_{c}$. However, unlike the FP-n based on past stress history and other classic state-dependent models [8,53,54], Equation (34) does not require an additional empirical state parameter, e.g., ψ, to capture the state dependence stress-dilatancy of soil, which is the main advantage of the FP-sn approach.

By considering the effect of the future reference critical state, a series of FP-sn models for modelling the state-dependent strength and deformation behaviour of granular soil and over-consolidated soft soil. Despite the positive model performance, there is still one problem with the FP-sn based on Equation (32): comparatively higher volumetric dilatancy of granular soil could be predicted due to the utilisation of the MCC function. As discussed before, the elastic region of the MCC surface at the ‘dry’ side of the critical state line is relatively large. A better model prediction can be obtained if using CC-based fractional dilatancy relation. However, it is difficult to analytically solve the fractional differentiations of the CC function, by incorporating the effect of future reference critical state. Further analytical work needs to be conducted.

## 3. FP-m: The Role of Past and Future Stress States

### Development of FP-m

In the previous section, two branches of the FP, i.e., FP-n: the one based on past SLS, and FP-sn: the other based on future reference critical state, were introduced. Even though each branch of the FP can be applied to describe various phenomenological behaviours of geomaterials, a question regarding the further comprehensive development of FP still rises: can one account for the roles of both past and future stress states, since they both can influence the plastic flow of geomaterials? Along with this consideration, we now modify the plastic flow rule by analogy with the FVP [15] to have a third definition of the FP, denoted as FP-m:

(38) $$d\varepsilon_{ij}^{p}=d\lambda_{\sigma_{ij}^{\prime }-l_{ij}}^{\;\;\;\;\;\;\;\;\mathrm{RC}}D_{\sigma_{ij}^{\prime }+{\tilde{l}}_{ij}}^{\alpha }f\left(\sigma_{ij}^{\prime }\right)$$

where $l_{ij}$ and ${\tilde{l}}_{ij}$ are the SLSs along the $\sigma_{ij}^{\prime }$–direction; the Riesz–Caputo fractional operator is adopted, such that

(39) $${}_{\sigma_{ij}^{\prime }-l_{ij}}^{\;\;\;\;\;\;\;\;\mathrm{RC}}D_{\sigma_{ij}^{\prime }+{\tilde{l}}_{ij}}^{\alpha }f\left(\sigma_{ij}^{\prime }\right)=\frac{1}{2}\left[{}_{\sigma_{ij}^{\prime }-l_{ij}}^{{}^{\;\;\;\;\;\;\;\;\;\;\;C}}D_{\sigma_{ij}^{\prime }}^{\alpha }f\left(\sigma_{ij}^{\prime }\right)+{\left(-1\right)}^{n}{}_{\sigma_{ij}^{{}^{\prime }}}^{\;\;\;\;\;C}D_{\sigma_{ij}^{\prime }+{\tilde{l}}_{ij}}^{\alpha }f\left(\sigma_{ij}^{\prime }\right)\right]$$

Substituting Equation (39) together with the MCC function into Equation (38), one can obtain the following generalised stress-dilatancy relation:

(40) $$d_{g}=M^{2}\frac{\left[l_{p}+\left(2-\alpha \right)\left(p^{\prime }-l_{p}-p_{0}^{\prime }/2\right)\right]l_{p}^{1-\alpha }+{\left(-1\right)}^{n}\left[{\tilde{l}}_{p}-\left(2-\alpha \right)\left(p^{\prime }+{\tilde{l}}_{p}-p_{0}^{\prime }/2\right)\right]{\tilde{l}}_{p}^{1-\alpha }}{\left[l_{q}+\left(2-\alpha \right)\left(q-l_{q}\right)\right]l_{q}^{1-\alpha }+{\left(-1\right)}^{n}\left[{\tilde{l}}_{q}-\left(2-\alpha \right)\left(q+{\tilde{l}}_{q}\right)\right]{\tilde{l}}_{q}^{1-\alpha }}$$

where $l_{p}$ and ${\tilde{l}}_{p}$ denote the long SLSs of the past and future stress states, respectively, along the $p^{{}^{\prime }}$–axis, while $l_{q}$ and ${\tilde{l}}_{q}$ denote the long SLSs of the past and future stress states, respectively, along with the *q*–axis. Through parameter analysis, one can find the following specific cases for Equation (40).

**Figure 12:**
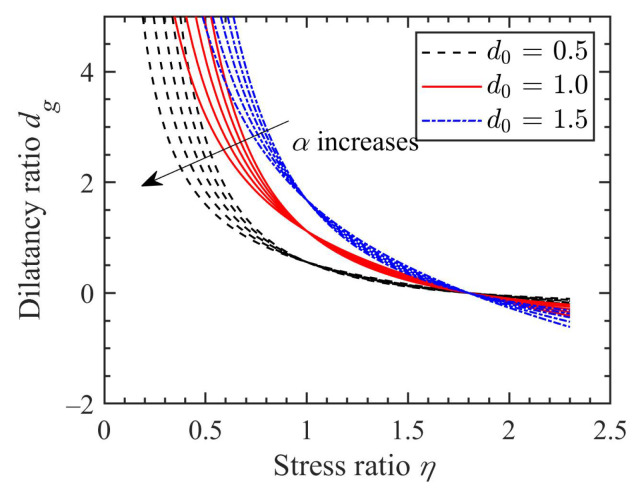
Effects of $d_{0}$ and α on the stress-dilatancy response.

• Case A
  It can be found that if α = 1 in Equation (40), then *n* = 1 and the stress-dilatancy relation reduces to the classic MCC-based one shown below, irrespective of $l_{p}$, ${\tilde{l}}_{p}$, $l_{q}$ and ${\tilde{l}}_{q}$.
  (41) $$d_{g}=\frac{M^{2}-\eta^{2}}{2\eta }$$
• Case B
  If one assumes that the SLSs of past and future stress states are equivalent, i.e., $l_{p}$ = ${\tilde{l}}_{p}$ and $l_{q}$ = ${\tilde{l}}_{q}$, then, the stress-dilatancy relation in Equation (40) can have two possible forms for α≠1. The first form can be obtained when α∈(0,1), which also indicates that *n* = 1. Thus, Equation (40) can be derived as:
  (42) $$d_{g}={\left(\frac{l_{p}}{l_{q}}\right)}^{1-\alpha }\frac{M^{2}-\eta^{2}}{2\eta }$$
  where it can be found that the future and past stress states contributes to the dilatancy of geomaterial by multiplying the original MCC-based dilatancy ratio with a factor of ${\left(l_{p}/l_{q}\right)}^{1-\alpha }$. Equation (42) can be further simplified by assuming that the SLSs, $l_{p}=xp^{\prime }$ and $l_{q}=y\eta p^{\prime }$, such that:
  (43) $$d_{g}=d_{0}\frac{M^{2}-\eta^{2}}{\eta^{2-\alpha }}$$
  where $d_{0}=1/2{\left(x/y\right)}^{1-\alpha }$, is a model parameter, indicating the upward or downward shifting of the dilatancy curve, as shown in Figure 12. With the increase of $d_{0}$, the dilatancy ratio at the same stress level increases. As α increases, the dilatancy ratio varies. Note that a similar empirical stress-dilatancy relation was also suggested for modelling crushable soil [55], which can be derived from Equation (42) by assuming a constant value of ${\left(l_{p}/l_{q}\right)}^{1-\alpha }$, e.g., ${\left(l_{p}/l_{q}\right)}^{1-\alpha }=M^{\alpha -1}$.

## 4. Conclusions

The FP was developed for modelling the state-dependent nonassociated constitutive behaviour of geomaterials. This study provided a comprehensive introduction and discussion on the development and application of the FP, from the perspective of the role of stress length scale. It can be found that three branches of the FP, i.e., FP-n, FP-sn and FP-m, can be defined, respectively, by considering the effects of past stress state and future reference critical state, or the impact of both past and future stress states. The advantages and disadvantages of each FP approach were discussed. Some main conclusions are summarized as follows:

- Based on the simulation results for geomaterials, the FP-n approach was found to be more effective than the associated flow rule. However, it is difficult for the original FP-n approach to consider state dependence unless an empirical state parameter was introduced. Hence, the FP-sn approach was developed to consider both state dependence and nonassociated plastic flow without using state parameter or additional plastic potential. Moreover, the FP-sn approach can predict a higher volumetric dilatancy of granular soil, due to its large elastic region at the ‘dry’ side of the critical state line.
- Further analytical work should be needed to propose a modified FP-sn approach by using a yielding surface with a reduced elastic region. Due to the dependence of both past and future stress states on material flow, the FP-m approach was also suggested, where several specific cases of the FP-m based dilatancy relation were discussed, with regard to the role of SLS.
- In future work, the fractional anisotropic damage model can be further studied based on the fractional plastic damage model mentioned in this paper. Moreover, combining with peridynamics and phase field methods, numerical implementation of fractional constitutive model will be an important research direction. By means of a physics-based deep neural network, fractional models can provide a novel sight for challenges faced in multiscale plasticity.

## Data Availability

Part of the data presented in this study are available on request from the corresponding author. The data are not publicly available due to intellectual property.

## Appendix A

Caputo’s definition of fractional derivatives is [56]:

(A1) $${}_{\;\;\;\;\;\sigma_{c}^{\prime }}^{\;\;\;\;\;\;\;C}D_{\sigma^{\prime }}^{\alpha }f\left(\sigma^{\prime }\right)=\frac{1}{\Gamma (n-\alpha )}\int_{\sigma_{c}^{\prime }}^{\sigma^{\prime }}\frac{f^{\left(n\right)}\left(\chi \right)d\chi }{{\left(\sigma^{\prime }-\chi \right)}^{\alpha +1-n}},\;\sigma^{\prime }>\sigma_{c}^{\prime }$$

(A2) $${}_{\sigma^{\prime }}^{\;\;C}D_{\sigma_{c}^{\prime }}^{\alpha }f\left(\sigma^{\prime }\right)=\frac{{\left(-1\right)}^{n}}{\Gamma (n-\alpha )}\int_{\sigma^{\prime }}^{\sigma_{c}^{\prime }}\frac{f^{\left(n\right)}\left(\chi \right)d\chi }{{\left(\chi -\sigma^{\prime }\right)}^{\alpha +1-n}},\;\sigma_{c}^{\prime }>\sigma^{\prime }$$

where Equation (A1) is the left-sided derivative while Equation (A2) is right-sided derivative; D (= $\partial^{\alpha }/\partial \sigma^{\prime \alpha }$) denotes the partial derivation of function *f*; Γ(x) is the gamma function and α∈(n−1,n), is the fractional order. $\sigma^{{}^{\prime }}$ and $\sigma_{c}^{{}^{\prime }}$ are the integral limits; χ is the independent variable.

The Riemann–Liouville’s fractional derivatives are [56]:

(A3) $${}_{0^{+}}^{\mathrm{RL}}D_{x}^{\alpha }f\left(x\right)=\frac{1}{\Gamma (n-\alpha )}\frac{d^{n}}{dx^{n}}\int_{0^{+}}^{x}\frac{f(\tau )d\tau }{{\left(x-\tau \right)}^{\alpha +1-n}},\;x>0$$

(A4) $${}_{x}^{\mathrm{RL}}D_{0^{-}}^{\alpha }f\left(x\right)=\frac{{\left(-1\right)}^{n}}{\Gamma (n-\alpha )}\frac{d^{n}}{dx^{n}}\int_{x}^{0^{-}}\frac{f(\tau )d\tau }{{\left(\tau -x\right)}^{\alpha +1-n}},\;x<0$$

The MCC loading criterion are adopted for modelling. Accordingly, the FP-m stress-dilatancy relation in Equation (40) can be revised as:

(A5) $$d_{g}=M^{2}\frac{\left[l_{p}+\left(2-\alpha \right)\left(p^{\prime }-l_{p}-p_{0}^{\prime }/2\right)\right]l_{p}^{1-\alpha }-\left[{\tilde{l}}_{p}-\left(2-\alpha \right)\left(p^{\prime }+{\tilde{l}}_{p}-p_{0}^{\prime }/2\right)\right]{\tilde{l}}_{p}^{1-\alpha }}{\left[l_{q}+\left(2-\alpha \right)\left(q-l_{q}\right)\right]l_{q}^{1-\alpha }-\left[{\tilde{l}}_{q}-\left(2-\alpha \right)\left(q+{\tilde{l}}_{q}\right)\right]{\tilde{l}}_{q}^{-\alpha }}$$

in which the assumptions in Case B are recalled to simplify Equation (A5).

## References

[1] Gao Y., Wu Y., Li D., Liu H., Zhang N. (2012). An improved approximation for the spectral representation method in the simulation of spatially varying ground motions. Probabilistic Eng. Mech..

[2] Zhang F., Gao Y., Wu Y., Zhang N. (2018). Upper-bound solutions for face stability of circular tunnels in undrained clays. Géotechnique.

[3] Been K., Jefferies M. (2004). Stress dilatancy in very loose sand. Can. Geotech. J..

[4] Been K., Jefferies M.G. (1985). A state parameter for sands. Géotechnique.

[5] Van Der Veen H., Vuik C., De Borst R. (1999). An eigenvalue analysis of nonassociated plasticity. Comput. Math. Appl..

[6] Lade P.V., Nelson R.B., Ito Y.M. (1987). Nonassociated flow and stability of granular materials. J. Eng. Mech..

[7] Yu H., Khong C., Wang J., Zhang G. (2005). Experimental evaluation and extension of a simple critical state model for sand. Granul. Matter.

[8] Shi X., Zhao J., Gao Y. (2021). A homogenization-based state-dependent model for gap-graded granular materials with fine-dominated structure. Int. J. Numer. Anal. Methods Geomech..

[9] Wood D.M. (1990). Soil Behaviour and Critical State Soil Mechanics.

[10] Ezzat M., El-Bary A. (2016). Unified fractional derivative models of magneto-thermo-viscoelasticity theory. Arch. Mech..

[11] Zenkour A., Abouelregal A. (2015). The fractional effects of a two-temperature generalized thermoelastic semi-infinite solid induced by pulsed laser heating. Arch. Mech..

[12] Raslan W. (2014). Application of fractional order theory of thermoelasticity to a 1D problem for a cylindrical cavity. Arch. Mech..

[13] Dinzart F., Lipiński P. (2009). Improved five-parameter fractional derivative model for elastomers. Arch. Mech..

[14] Sumelka W. (2014). A note on non-associated Drucker-Prager plastic flow in terms of fractional calculus. J. Theor. Appl. Mech..

[15] Sumelka W. (2014). Fractional viscoplasticity. Mech. Res. Commun..

[16] Sun Y., Sumelka W. (2021). Multiaxial stress-fractional plasticity model for anisotropically overconsolidated clay. Int. J. Mech. Sci..

[17] Lu D., Liang J., Du X., Ma C., Gao Z. (2019). Fractional elastoplastic constitutive model for soils based on a novel 3D fractional plastic flow rule. Comput. Geotech..

[18] Lu D., Zhou X., Du X., Wang G. (2019). A 3D fractional elastoplastic constitutive model for concrete material. Int. J. Solids Struct..

[19] Qu P., Zhu Q., Sun Y. (2019). Elastoplastic modelling of mechanical behavior of rocks with fractional-order plastic flow. Int. J. Mech. Sci..

[20] Qu P., Zhu Q., Zhao L., Cao Y. (2021). A micromechanics-based fractional frictional damage model for quasi-brittle rocks. Comput. Geotech..

[21] Sumelka W., Nowak M. (2016). Non-normality and induced plastic anisotropy under fractional plastic flow rule: A numerical study. Int. J. Numer. Anal. Methods Geomech..

[22] Sumelka W., Nowak M. (2018). On a general numerical scheme for the fractional plastic flow rule. Mech. Mater..

[23] Perzyna P. (1963). The constitutive equations for rate sensitive plastic materials. Q. Appl. Math..

[24] Sun Y., Shen Y. (2017). Constitutive model of granular soils using fractional-order plastic-flow rule. Int. J. Geomech..

[25] Sun Y., Gao Y., Zhu Q. (2018). Fractional order plasticity modelling of state-dependent behaviour of granular soils without using plastic potential. Int. J. Plast..

[26] Le L.A., Nguyen G.D., Bui H.H., Andrade J.E. (2021). Modelling the influence of fines content on the instability of silty sands considering grain scale interactions. Int. J. Plast..

[27] Nguyen H., Rahman M., Fourie A. (2020). Effect of particle shape on constitutive relation: DEM study. J. Geotech. Geoenvironmental Eng..

[28] Nguyen H.B.K., Rahman M.M., Fourie A. (2021). The critical state behaviour of granular material in triaxial and direct simple shear condition: A DEM approach. Comput. Geotech..

[29] Sun Y., Gao Y., Shen Y. (2019). Mathematical aspect of the state-dependent stress–dilatancy of granular soil under triaxial loading. Géotechnique.

[30] Polizzotto C. (2001). Nonlocal elasticity and related variational principles. Int. J. Solids Struct..

[31] Patnaik S., Semperlotti F. (2020). A generalized fractional-order elastodynamic theory for non-local attenuating media. Proc. R. Soc. A.

[32] Sun Y., Gao Y., Song S. (2018). Effect of integrating memory on the performance of the fractional plasticity model for geomaterials. Acta Mech. Sin..

[33] Sun Y., Xiao Y. (2017). Fractional order plasticity model for granular soils subjected to monotonic triaxial compression. Int. J. Solids Struct..

[34] Wu L., Cheng W., Zhu Z. (2021). Fractional-Order elastoplastic modeling of sands considering cyclic mobility. J. Mar. Sci. Eng..

[35] Zhang T., Zhou X., Qian Q. (2022). The peridynamic Drucker-Prager plastic model with fractional order derivative for the numerical simulation of tunnel excavation. Int. J. Numer. Anal. Methods Geomech..

[36] Rahman M.M., Lo S.C., Dafalias Y. (2014). Modelling the static liquefaction of sand with low-plasticity fines. Géotechnique.

[37] Nguyen G.T., Chan E.L., Tsuji T., Tanaka T., Washino K. (2021). Resolved CFD–DEM coupling simulation using Volume Penalisation method. Adv. Powder Technol..

[38] Schofield A.N., Wroth P. (1968). Critical State Soil Mechanics.

[39] Liang J., Lu D., Zhou X., Du X., Wu W. (2019). Non-orthogonal elastoplastic constitutive model with the critical state for clay. Comput. Geotech..

[40] Liang J., Lu D., Du X., Wu W., Ma C. (2020). Non-orthogonal elastoplastic constitutive model for sand with dilatancy. Comput. Geotech..

[41] Verdugo R., Ishihara K. (1996). The steady state of sandy soils. Soils Found..

[42] Sun Y., Sumelka W., Gao Y. (2020). Bounding surface plasticity for sand using fractional flow rule and modified critical state line. Arch. Appl. Mech..

[43] Sun Y., Nimbalkar S. (2019). Stress-fractional soil model with reduced elastic region. Soils Found..

[44] Sun Y., Sumelka W., He S., Gao Y. (2022). Enhanced Fractional Model for Soil–Structure Interface Considering 3D Stress State and Fabric Effect. J. Eng. Mech..

[45] Wu E., Zhu J., Sun Y., He S. (2022). A general plastic model for rockfill material developed by using Caputo fractional derivative. Comput. Geotech..

[46] Li H., Ma B., Zhang S., Sheng D. (2020). Mechanical behaviors of soft rocks based on the fractional thermal elastic-plastic theory. Chin. J. Rock Mech. Eng..

[47] Shen W., Liu S., Xu W., Shao J. (2022). An elastoplastic damage constitutive model for rock-like materials with a fractional plastic flow rule. Int. J. Rock Mech. Min. Sci..

[48] Qu P.F., Zhu Q.Z. (2021). A Novel Fractional Plastic Damage Model for Quasi-brittle Materials. Acta Mech. Solida Sin..

[49] Zhou X., Lu D., Du X., Wang G., Meng F. (2020). A 3D non-orthogonal plastic damage model for concrete. Comput. Methods Appl. Mech. Eng..

[50] Lu D., Su C., Zhou X., Wang G., Du X. (2022). A cohesion-friction combined hardening plastic model of concrete with the nonorthogonal flow rule: Theory and numerical implementation. Constr. Build. Mater..

[51] Halilovič M., Vrh M., Štok B. (2009). NICE—An explicit numerical scheme for efficient integration of nonlinear constitutive equations. Math. Comput. Simul..

[52] Zhu Q., Zhao L., Shao J. (2016). Analytical and numerical analysis of frictional damage in quasi brittle materials. J. Mech. Phys. Solids.

[53] Li X.S., Dafalias Y.F. (2000). Dilatancy for cohesionless soils. Géotechnique.

[54] Dafalias Y.F., Manzari M.T. (2004). Simple plasticity sand model accounting for fabric change effects. J. Eng. Mech..

[55] Liu M., Gao Y. (2017). Constitutive modeling of coarse-grained materials incorporating the effect of particle breakage on critical state behavior in a framework of generalized plasticity. Int. J. Geomech..

[56] Zhou Y. (2015). Fractional evolution equations and inclusions. Analysis and Control.

